# Supplementary material for: Evolution of the murine gut resistome following broad-spectrum antibiotic treatment
Source: Nat Commun. 2022 Apr 28;13:2296. doi: 10.1038/s41467-022-29919-9 (PMC9051133; doi:10.1038/s41467-022-29919-9)
Supplement: Supplementary file 1 — Supplementary Information [file 41467_2022_29919_MOESM1_ESM.pdf]

Supplementary Information: Evolution of the murine gut resistome following broad-spectrum antibiotic treatment

Laura de Nies[#,1], Susheel Bhanu Busi[#,1], Mina Tsenkova[2], Rashi Halder[1], Elisabeth Letellier[2,*] and Paul Wilmes[1,2,*]

[1]Systems Ecology Group, Luxembourg Centre for Systems Biomedicine, University of Luxembourg, Esch-sur-Alzette, Luxembourg
[2]Department of Life Sciences and Medicine, Faculty of Science, Technology and Medicine, University of Luxembourg, Esch-sur-Alzette, Luxembourg

[#]Equal contribution

Running title: Integrons mediate AMR under selective antibiotic pressure

*Corresponding authors: Paul Wilmes (*paul.wilmes@uni.lu*) and Elisabeth Letellier (*elisabeth.letellier@uni.lu*)

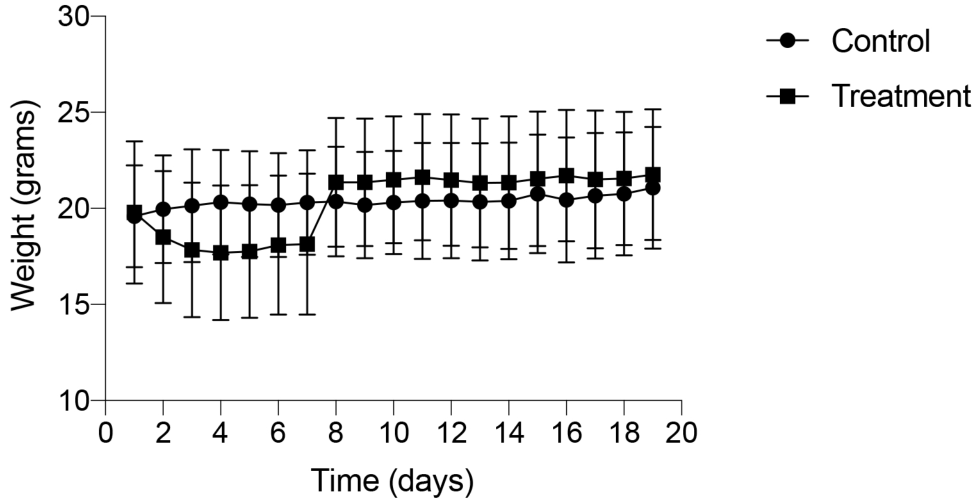

Supplementary figure 1. Physiological characterisation of mice across timepoints
Daily weight measurements throughout the experimental duration. Bars indicate standard deviation (±SD). n=8 biological replicates per group. Source data are provided as a Source Data file.

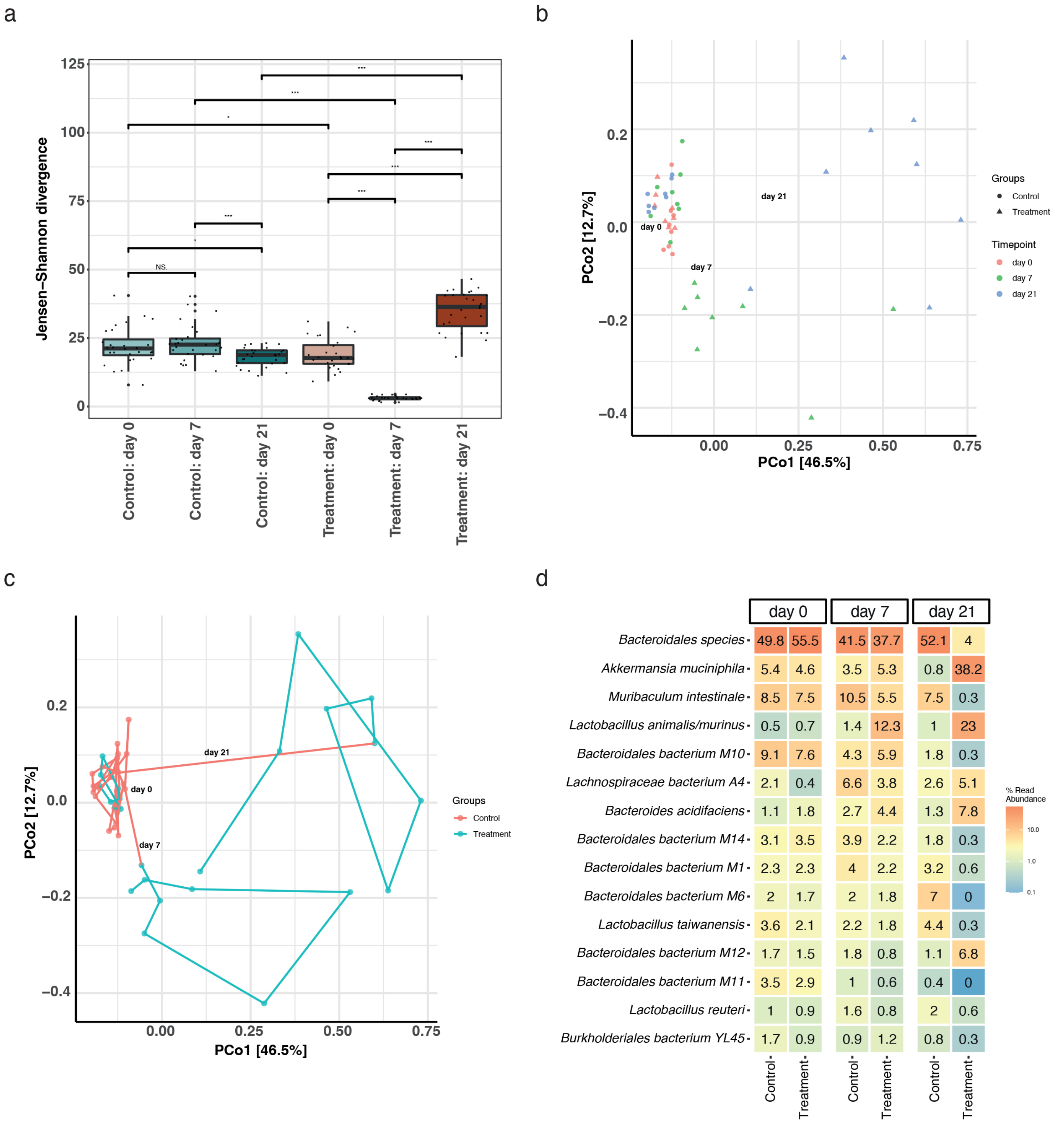

Supplementary figure 2. Depletion of taxa after antibiotic treatment

a) Jensen-Shannon Divergence (JSD) estimates of the microbial community for the control and treatment groups (n=8 biological replicates) at days 0, 7 and 21. Two-way ANOVA was used to assess significance, where, adjusted p-values are indicated by *, i.e., * < 0.05, ** < 0.01, *** < 0.001. The centre line denotes the median value (50th percentile), while the outer lines of the box represent the 25th to 75th percentiles. The black whiskers mark the 5th and 95th percentiles. Significance was assessed using a two-sided Wilcoxon rank sum test, where, adjusted p-values are indicated by *, i.e., * < 0.05, ** < 0.01, *** < 0.001. b) Principal component analyses generated from metagenomic operational taxonomic unit (mOTU) profiles at the Genus level for the control and treatment groups at days 0, 7 and 21. c) Time-tracked ordination plot representing the overall changes in community profile between day 0 through day 7 to day 21. Significance was assessed using Permutational Multiple Analysis of Variance (PERMANOVA). d) Relative abundance of the significantly different mOTUs (adj. p < 0.05, Two-way ANOVA) in the control and treatment groups at different timepoints. Source data are provided as a Source Data file.

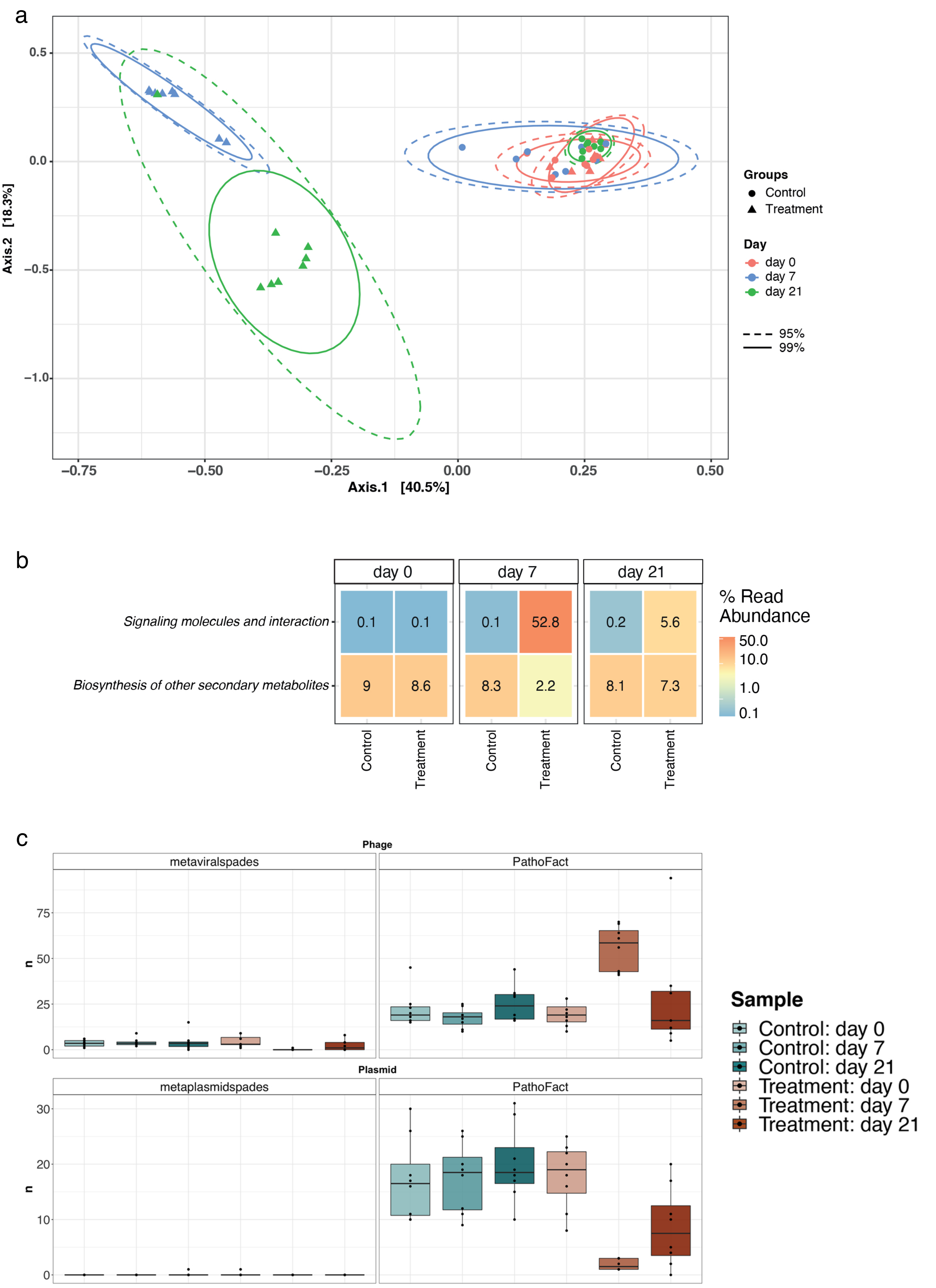

Supplementary figure 3. Differential KEGG pathway and MGE analysis

a) Time-tracked ordination plot of the metagenomic functional profile indicating the changes of the KEGG ortholog functions across time between the control and antibiotic-treated mice. Dashed and solid lines indicate the 95% and 99% confidence intervals, where the significant differences between the groups (n=8 biological replicates) was assessed using a PERMANOVA. b) KEGG pathways that are significantly different (adj. $p < 0.05$, Two-way ANOVA) between the treatment and control groups, are shown as a heatmap displaying longitudinal changes. c) Boxplots depicting the number of AMR-associated contigs (n) on the y-axis found when assessed using metaPLASMIDspades, metaVIRALspades and PathoFact. The centre line denotes the median value (50th percentile), while the outer lines of the box represent the 25th to 75th percentiles. The black whiskers mark the 5th and 95th percentiles. Source data are provided as a Source Data file.

a

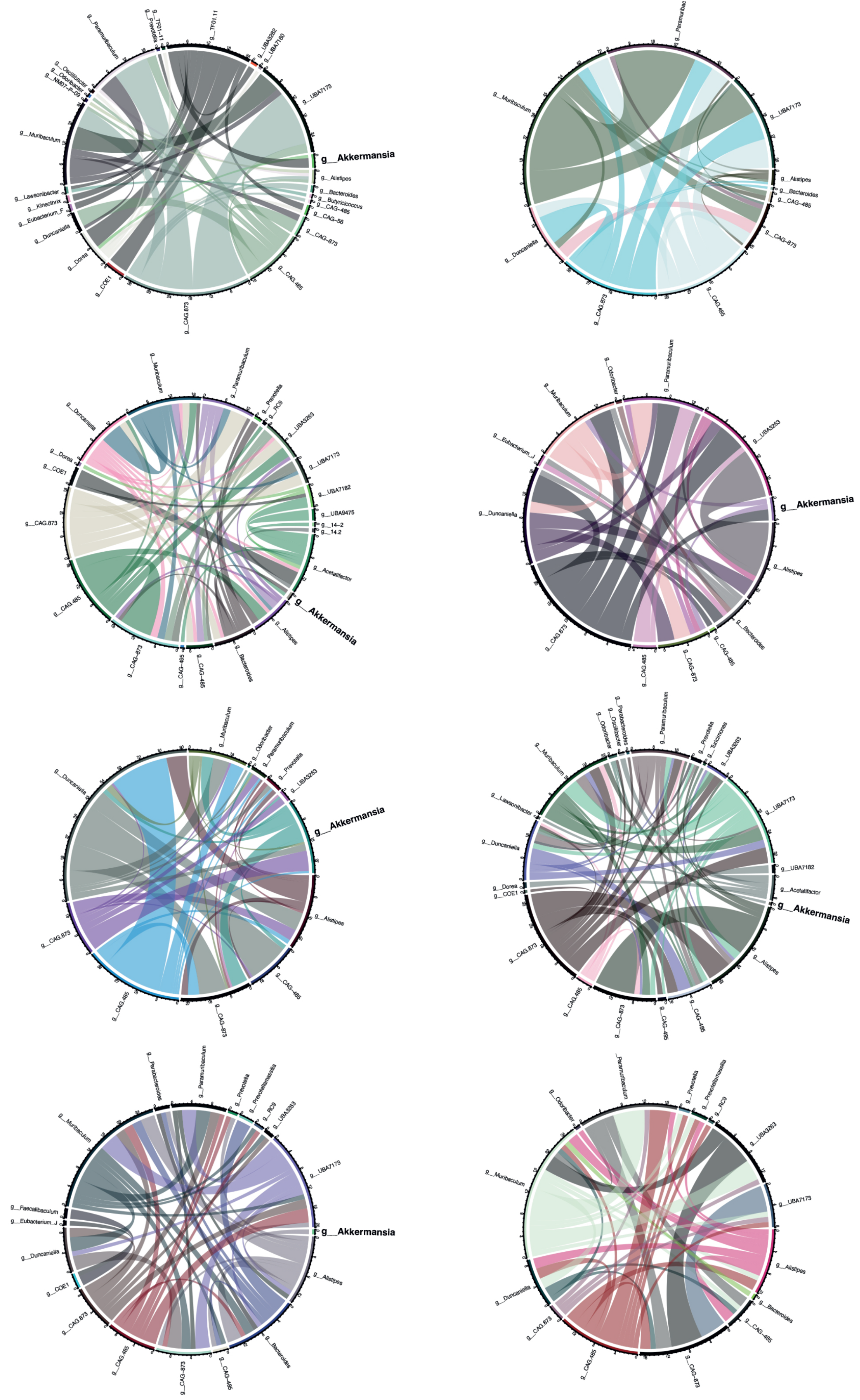
g__Akkermansia
g__Alistipes
g__Bacteroides
g__Butyricicoccus
g__CAG-485
g__CAG-56
g__CAG-873
g__CAG.485
g__CAG.873
g__COE1
g__Dorea
g__Duncaniella
g__Eubacterium_F
g__Kineothrix
g__Lawsonibacter
g__Muribaculum
g__NM07-P-09
g__Odoribacter
g__Oscillibacter
g__Paramuribaculum
g__Prevotella
g__TF01-11
g__TF01.11
g__UBA3282
g__UBA7160
g__UBA7173
g__Paramuribac
g__RC9
g__UBA3263
g__UBA7182
g__UBA9475
g__14-2
g__14.2
g__Acetatifactor
g__Eubacterium_J
g__Parabacteroides
g__Turicimonas
g__Faecalibaculum
g__Prevotellamassilia

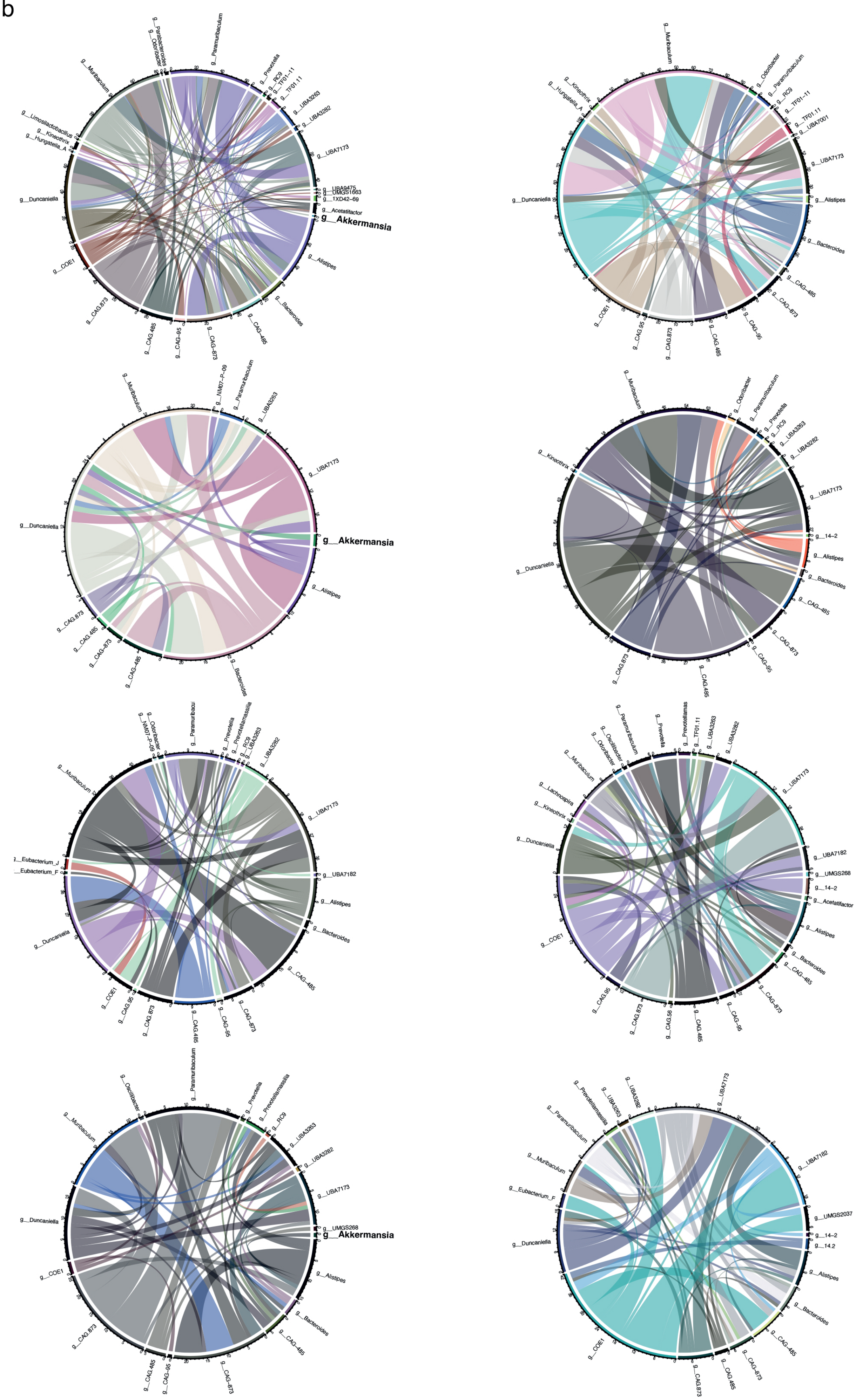

Supplementary figure 4. Horizontal gene transfer events
a) MetaCHIP-based analysis of HGT events between Akkermansia muciniphila and the remaining MAGs in the control samples. b) HGT events between Akkermansia muciniphila and the remaining MAGs in the mice treated with antibiotics.

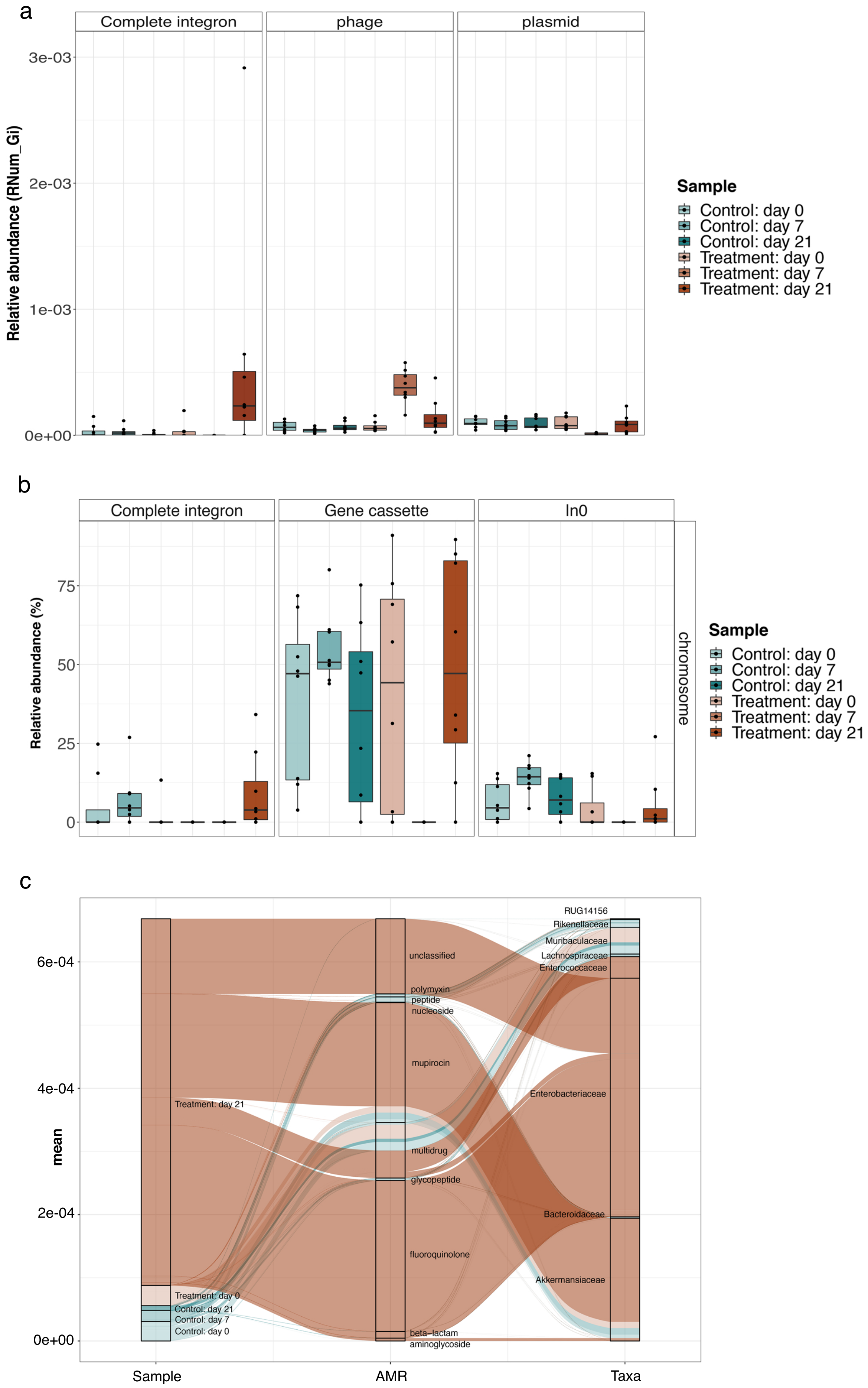

Supplementary figure 5. Integron-derived AMR

a) Boxplots indicating the relative abundance of complete integrons found across all timepoints in comparison with complete integrons linked to phages and plasmids. n=8 biological replicates per group. The centre line denotes the median value (50th percentile), while the outer lines of the box represent the 25th to 75th percentiles. The black whiskers mark the 5th and 95th percentiles. b) Boxplots depicting the relative abundance of gene cassettes, complete and incomplete integrons (In0) linked to ARGs found on the bacterial chromosome across the control and treatment groups (n=8 biological replicates) at all timepoints. The centre line denotes the median value (50th percentile), while the outer lines of the box represent the 25th to 75th percentiles. The black whiskers mark the 5th and 95th percentiles. c) Alluvial plot indicating integron-mediated AMR categories at all timepoints and the corresponding taxa they are associated with. Source data are provided as a Source Data file.

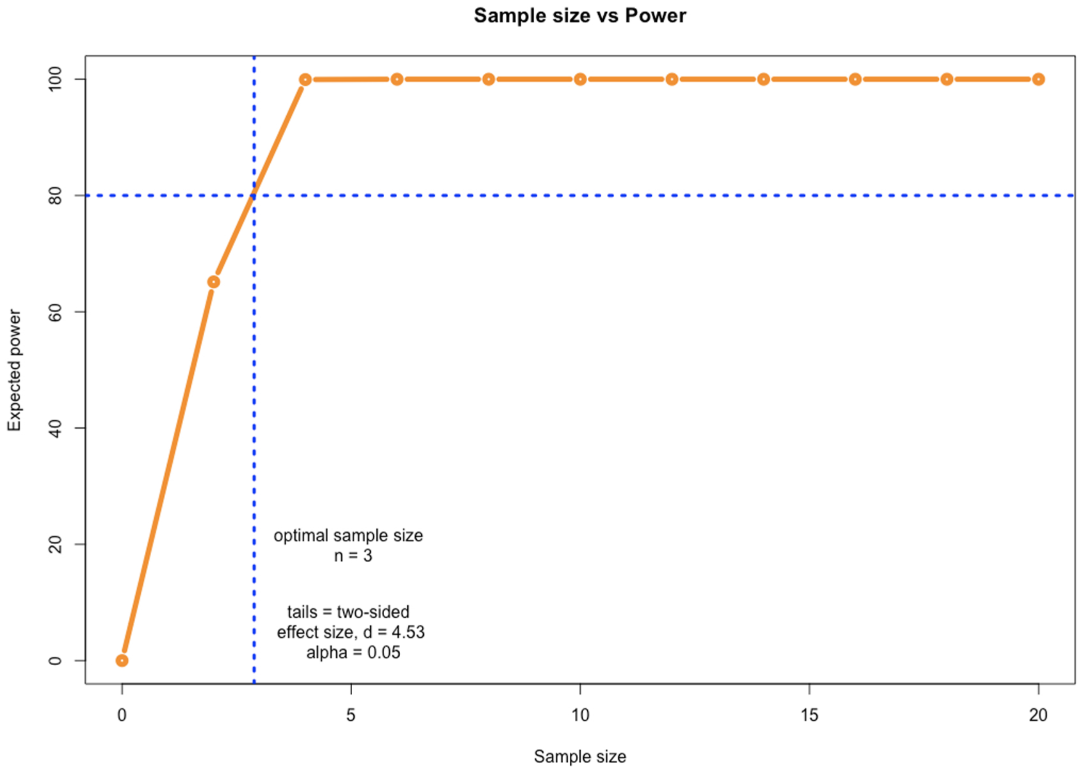

Supplementary figure 6. Sample size estimation

Sample size estimate measured using Jensen-Shannon Divergences between control and antibiotic-treated samples based on a study by Raymond et al.51. The required sample for an estimated Type I (alpha) error rate of 5% and a power of 80% was determined accordingly.
