# Supplementary material for: Evolution of the murine gut resistome following broad-spectrum antibiotic treatment
Source: Nat Commun. 2022 Apr 28;13:2296. doi: 10.1038/s41467-022-29919-9 (PMC9051133; doi:10.1038/s41467-022-29919-9)
Supplement: Supplementary file 3 — Reporting Summary [file 41467_2022_29919_MOESM3_ESM.pdf]

nature research

Corresponding author(s): Paul Wilmes and Elisabeth Letellier; NCOMMS-21-04264B

Last updated by author(s): Apr 1, 2022

Reporting Summary

Nature Research wishes to improve the reproducibility of the work that we publish. This form provides structure for consistency and transparency in reporting. For further information on Nature Research policies, see our Editorial Policies and the Editorial Policy Checklist.

Statistics

For all statistical analyses, confirm that the following items are present in the figure legend, table legend, main text, or Methods section.

n/a	Confirmed	
☐	☒	The exact sample size (*n*) for each experimental group/condition, given as a discrete number and unit of measurement
☐	☒	A statement on whether measurements were taken from distinct samples or whether the same sample was measured repeatedly
☐	☒	The statistical test(s) used AND whether they are one- or two-sided<br>*Only common tests should be described solely by name; describe more complex techniques in the Methods section.*
☒	☐	A description of all covariates tested
☐	☒	A description of any assumptions or corrections, such as tests of normality and adjustment for multiple comparisons
☐	☒	A full description of the statistical parameters including central tendency (e.g. means) or other basic estimates (e.g. regression coefficient) AND variation (e.g. standard deviation) or associated estimates of uncertainty (e.g. confidence intervals)
☐	☒	For null hypothesis testing, the test statistic (e.g. F, t, r) with confidence intervals, effect sizes, degrees of freedom and P value noted<br>*Give P values as exact values whenever suitable.*
☒	☐	For Bayesian analysis, information on the choice of priors and Markov chain Monte Carlo settings
☒	☐	For hierarchical and complex designs, identification of the appropriate level for tests and full reporting of outcomes
☒	☐	Estimates of effect sizes (e.g. Cohen's d, Pearson's r), indicating how they were calculated

Our web collection on statistics for biologists contains articles on many of the points above.

Software and code

Policy information about availability of computer code

Data collection	The open-source tools and algorithms used for the data analyses are reported in the Methods section, including relevant flags used for the various tools. The scripts and analysis codes are provided at https://git-r3lab.uni.lu/susheel.busi/intonate.<br><br>Code availability<br>The open-source tools and algorithms used for the data analyses are reported in the Methods section, including relevant flags used for the various tools. The scripts and analysis codes are provided at https://git-r3lab.uni.lu/susheel.busi/intonate.
Data analysis	Data processing for metagenomics, including genome reconstruction<br>The Integrated Meta-omic Pipeline (IMP; v3 - commitID #6f1badf7) was used to process paired forward and reverse reads using the built-in metagenomic workflow as previously described55. The workflow includes pre-processing, assembly, genome reconstruction and functional annotation of genes based on custom databases in a reproducible manner. After trimming the adapters, the preprocessing step included the removal of Mus musculus (GRCm38.p6 (GCA_000001635.8); retrieved on 16-May-2020 from https://www.ensembl.org/Mus_musculus/Info/Index) reads. Thereafter the de novo assembly was performed using the MEGAHIT (version 2.0) assembler. Default IMP parameters were retained for all samples. Metagenomic operational taxonomic unit (mOTU) profiles were generated from the trimmed and preprocessed reads to generate microbiome profiles for the control and treatment groups using mOTUs v2.5.1. Concurrently, we used MetaBAT2 and MaxBin2 for binning in addition to an in-house binning methodology previously described55 for genome reconstructions, i.e. metagenome-assembled genomes (MAGs). Subsequently, we obtained a non-redundant set of MAGs using DASTool v1.1.4 with a score threshold of 0.7 for downstream analyses, and those with a minimum completion of 90% and less than 5% contamination as assessed by CheckM v1.1.3. Taxonomy was assigned to the MAGs using the extensive database packaged with gtdbtk v1.7.0. To generate pangenomes, we collected all the bins taxonomically identified as Akkermansia muciniphila and used the anvi'o-based pangenome workflow described by Eren et. al (http://merenlab.org/2016/11/08/pangenomics-v2/). One of the treated mice (#16), was excluded from the pangenome analyses due to the unavailability of MAGs.

Identification of antimicrobial resistance genes and association with mobile genetic elements
We used PathoFact v1.0, a pipeline for the prediction of virulence factors and antimicrobial resistance genes, to predict and identify ARGs within our metagenomes. The assembly files from individual samples were used as input for the AMR analyses. To assess the relevance of metaplasmidSPAdes and metaviralSPAdes for identifying plasmid and viral sequences respectively, we used de novo SPAdes assembler v3.15.4. For the assembly, we used the same kmer settings as the assembly setup in IMP, i.e. 21, 33, 55, and 77, in a paired-end format with 24 threads. Subsequently, ARGs were collapsed into categories based on the Comprehensive Antibiotic Resistance Database (CARD) and identified using PathoFact. Thereafter, the relative abundance of the ARGs was calculated using the Rnum_Gi method described by Hu et al.

Identified ARGs and their categories were linked to associated bacterial taxonomy using the metagenomic bin classifications. Furthermore, utilizing PathoFact, ARGs were linked to predicted mobile genetic elements (MGEs: phages and plasmids) to identify probable transmission of AMR between taxa. More specifically, to link both the MGEs and the taxonomy to the ARGs, we mapped the genes to assembled contigs, followed by identifying the corresponding bins (MAGs) to which the contigs belonged. By considering all different predictions of MGEs, a final classification was made based on the genomic contexts of the ARGs encoded on plasmids, phages or chromosomes, including classification of those that could not be resolved (ambiguous). The ARGs that could not be assigned to either the MGEs or bacterial chromosomes were further referred to as unclassified genomic elements. Certain ARGs were encoded on both the bacterial chromosome and phage genomes. HGT of ARGs was assessed using MetaCHIP v1.0 with a modified setting of full-length match of genes of interest to ensure robustness of the findings. The confirmation of ARGs and their associated mode of transfer was also performed manually alongside this by mapping identical 1Kbp flanking regions, via the same pipeline. Briefly, groups of genes among all input MAGs with maximum average identity were considered putative HGT genes. To validate the predicted candidates, a pairwise BLASTN was used to assess each pair of flanking regions of 10 Kbp. Visual representations of the genomic regions were extracted alongside the results for visual interpretation and inspection.

Linking antimicrobial resistance genes with integrons
The assemblies generated via IMP were used to assess the presence and abundance of integrons within the metagenomes. Briefly, attC sites were identified by HattCI while for the annotation of the intI sites a BLAST database was created using the intI variant sequences from the UniProt database. Only those contigs where both the signature genetic regions (intI and attC) were found were annotated as having 'complete' integron elements. We also identified the MAGs along with which the integrons were binned, thus linking the integrons to the reconstructed genomes. The ARG information was overlaid onto this to identify contigs where integrons were linked with ARGs. Furthermore, we used sequence coordinates to identify integron localization, i.e., chromosome, plasmid or phage localization of gene cassettes, incomplete and complete integrons on MGEs. This information was used for downstream differential analyses.

Data analysis
Figures for the study including visualizations derived from the taxonomic and functional, were created using version 3.6 of the R statistical software package. GraphPad was used to generate the figures for describing the longitudinal weight measurements of the mice. DESeq2 and Wilcoxon rank-sum tests with FDR-adjustments for multiple testing were used to assess significant differences for the AMR and taxonomic analyses whereas a paired two-way ANOVA (Analysis of Variance) within the nlme package was used for identifying statistically significant differences in the integron profiles. Chord diagrams for the HGT events were generated using scripts found within the MetaCHIP package while the pangenome visualizations were obtained using anvi'o.

For manuscripts utilizing custom algorithms or software that are central to the research but not yet described in published literature, software must be made available to editors and reviewers. We strongly encourage code deposition in a community repository (e.g. GitHub). See the Nature Research guidelines for submitting code & software for further information.

Data

Policy information about availability of data
All manuscripts must include a data availability statement. This statement should provide the following information, where applicable:
- Accession codes, unique identifiers, or web links for publicly available datasets
- A list of figures that have associated raw data
- A description of any restrictions on data availability

The sequencing data generated for this study are available via NCBI's Sequence Read Archive under the accession number: PRJNA691897. The metadata file indicating group and timepoint information can be obtained via the same accession ID.

To determine the number of animals required per treatment and control group we performed a multifactorial power analysis based on a 2015 study (PMID: 24445449). The data from this study was used in the analyses for power calculation.

For trimming mouse reads the Mus musculus (GRCm38.p6 (GCA_000001635.8) data was used; retrieved on 16-May-2020 from https://www.ensembl.org/Mus_musculus/Info/Index) reads.

Additionally, the CARD database (https://card.mcmaster.ca/) for antimicrobial resistance genes was used.

Field-specific reporting

Please select the one below that is the best fit for your research. If you are not sure, read the appropriate sections before making your selection.

☒ Life sciences ☐ Behavioural & social sciences ☐ Ecological, evolutionary & environmental sciences

For a reference copy of the document with all sections, see nature.com/documents/nr-reporting-summary-flat.pdf

Life sciences study design

All studies must disclose on these points even when the disclosure is negative.

Sample size	Based on a previous study (PMID: 24445449), the pairwise distances in gut microbiota diversity between Antibiotic-treated and control group animals was estimated using the Jensen-Shannon index. Using this metric, a sample size calculation revealed a minimum number of 3 mice per group to achieve a power of 80% with a significance threshold of 5% given the drastic shift in diversity post-antibiotic treatment.
Data exclusions	No data were excluded from the study.
Replication	The experiment was performed independently, where each individual mouse within the treatment and control groups serve as a 'biological replicate' for the treatment protocol. Each mouse was housed independently ensuring robustness of our findings. Given the consistency in the observed results with respect to both gut microbiota profiles and also plasmid-borne integron-mediated horizontal gene transfer of AMR, we believe our findings are rigorous and reproducible.
Randomization	Mice were randomly distributed into separate cages post-weaning and subsequently assigned to individual groups on a random basis by an animal technician not directly involved with the study.
Blinding	To avoid bias with respect to differential handling of mice, a single person was designated for the animal work and thus blinding was not possible. The data analyses were however performed in a single-blind fashion, mapping the sample groups to the individual mice towards the end of the downstream analyses.

Reporting for specific materials, systems and methods

We require information from authors about some types of materials, experimental systems and methods used in many studies. Here, indicate whether each material, system or method listed is relevant to your study. If you are not sure if a list item applies to your research, read the appropriate section before selecting a response.

Materials & experimental systems

n/a	Involved in the study	
☒	☐	Antibodies
☒	☐	Eukaryotic cell lines
☒	☐	Palaeontology and archaeology
☐	☒	Animals and other organisms
☒	☐	Human research participants
☒	☐	Clinical data
☒	☐	Dual use research of concern

Methods

n/a	Involved in the study	
☒	☐	ChIP-seq
☒	☐	Flow cytometry
☒	☐	MRI-based neuroimaging

Animals and other organisms

Policy information about studies involving animals; ARRIVE guidelines recommended for reporting animal research

Laboratory animals	C57BL/6J mice were bred in-house and experiments were performed according to all applicable laws and the regulations, after receiving approval by the institution's animal experimentation ethics committee and the veterinarian service of the Ministry of Agriculture (Permit Number: LUPA 2019/13). To limit individual variation of the gut microbiome in experimental groups, mice of the same age were obtained from the same vendor and the same location in the vendor facility. After a 7-day quarantine and subsequent acclimation period of one week, mice were maintained in single housing conditions for each experiment. Mice were housed in Allentown NexGen Mouse 500 (194mm x 130mm x 381 mm) cages with JRS Rehofix Corncob bedding. Mice had access to reverse osmosis water with 2ppm of chlorine fed ad libitum along with standard A40 chow diet (SAFE, France). The animals were maintained under standard habitat conditions (humidity: 40-70%, temperature: 22°C) with a 12:12 light cycle. Two groups of mice were established (control and treatment), and each group contained 4 animals (2 males + 2 females). Antibiotics, ampicillin (1g/L), vancomycin (500mg/L), metronidazole (1g/L) and neomycin (1g/L) were chosen for their utility in several mouse models58,59 and in line with most preoperative procedures60. They were administered as a cocktail within the drinking water to the treatment group starting at 8 weeks of age. Antibiotics were administered during a period of one week, after which the change was made to regular drinking water for the duration of the recovery period. Fecal samples were collected daily for a duration of 19 days (both treatment and recovery phase) starting prior to the antibiotic treatment till take down
Wild animals	No wild animals were used in the study.
Field-collected samples	The study did not involve samples collected from the field
Ethics oversight	University of Luxembourg's animal experimentation ethics committee and the veterinarian service of the Luxembourg Ministry of Agriculture (Permit Number: LUPA 2019/13).<br><br>Names of the approving entites: University of Luxembourg and Luxembourg Ministry of Agriculture

Note that full information on the approval of the study protocol must also be provided in the manuscript.
